# How the CIOMS guidelines contribute to fair inclusion of pregnant women in research

**DOI:** 10.1111/bioe.12520

**Published:** 2018-10-20

**Authors:** Rieke van der Graaf, Indira S. E. van der Zande, Johannes J. M. van Delden

**Affiliations:** ^1^ Julius Center for Health Sciences and Primary Care University Medical Center Utrecht Utrecht Netherlands; ^2^ Medical Centre Utrecht University Utrecht Netherlands

**Keywords:** CIOMS guidelines, learning health systems, pregnant women, research ethics, vulnerability

## Abstract

As early as 2002, CIOMS stated that pregnant women should be presumed eligible for participation in research. Despite this position and calls of other well‐recognized organizations, the health needs of pregnant women in research remain grossly under‐researched. Although the presumption of eligibility remains unchanged, the revision of the 2002 CIOMS *International ethical guidelines for biomedical research involving human subjects* involved a substantive rewrite of the guidance on research with pregnant women and related guidelines, such as those on fair inclusion and vulnerability. However, close reading of the guidelines reveals morally relevant different approaches to fair inclusion of pregnant women and other under‐represented groups, such as children and incompetents. Where CIOMS sets out that children and adolescents must be included unless a good scientific reason justifies their exclusion, no such claim of having to justify exclusion appears in the guideline on pregnant women. Instead, CIOMS claims that research relevant to pregnant women’s health needs must be promoted. This paper analyses how and to what extent the guideline on pregnant women differs from other guidance on fair inclusion in the document. Accordingly, the paper evaluates to what extent the current phrasing may contribute to fair inclusion of pregnant women in research. We will conclude that a system change towards a learning health system is essential to break down the status quo of knowledge generation in the field of medication use during pregnancy and argue that the CIOMS guidelines allow for this system change.

## 1 INTRODUCTION

Historically, there has been a reluctance to include pregnant women in research due to a fear of harming the fetus. Often mentioned in this respect are the diethylstilboestrol (DES) and thalidomide tragedies. From 1938 to 1971, DES was prescribed to an estimated 1.5 to 3 million women during pregnancy to prevent miscarriage. The drug turned out not to prevent miscarriage and was linked to several adverse complications for the offspring, including vaginal and cervical carcinomas in young women, and malformation of reproductive organs in both male and female children.^1^ In the late 1950s, thalidomide was prescribed for nausea during pregnancy without prior testing in pregnant women, which resulted in unforeseen teratogenic effects with severe birth defects in over 10,000 children.^2^ Even though neither tragedy involved clinical research, these events had a great impact on the research community, which at the time was already characterized by a determination to protect allegedly vulnerable populations, including pregnant women, from research participation. The protectionist approach is one of the reasons for the existing precautionary attitude with regard to including pregnant women in clinical research today.^3^

Although concerns about fetal well‐being are valid, at the same time there is a need for evidence‐based information on medications and treatments for pregnant women, because ‘pregnant women get sick, and sick women get pregnant’.^4^ In the absence of evidence‐based knowledge, clinicians may have to prescribe off‐label medications without evidence or based on contradictory evidence,^5^ or clinicians or pregnant women themselves may choose to discontinue medically important medications.^6^ The result of under‐representation of pregnant women in clinical research is a harmful situation, leaving pregnant women at risk for potentially avoidable therapeutic incidents.^7^ For example, poorly treated asthma and untreated depression are problematic for pregnant women and fetuses: both are associated with premature birth, low birthweight and fetal growth restriction and, in the case of asthma, a higher risk of hypertension and pre‐eclampsia.^8^

Physiological changes during pregnancy alter the way that drugs are processed by the body and the ways that drugs act on the body in a fashion difficult to predict from the pharmacokinetics and pharmacodynamics in men and non‐pregnant women. Moreover, information about teratology and toxicity is often difficult to extrapolate and interpret from preclinical data or studies in non‐pregnant humans.^9^ Gathering conclusive data to enable evidence‐based therapeutic decisions for pregnant women therefore requires research in the population of pregnant women in order to develop effective treatments for pregnant women with acute or chronic obstetric and non‐obstetric illnesses.^10^

Inclusion of pregnant women in research has been promoted in the last decades by, among others, medical ethicists, pharmacologists, regulators and researchers. For example, in the United States, the Office of Research on Women’s Health (ORWH) of the Department of Health and Human Services (DHHS) endorsed the view that pregnant women are to be presumed eligible for participation in clinical research (1994). This view was later adopted by the Council for International Organizations of Medical Sciences (CIOMS) in their *International ethical guidelines for biomedical research involving human subjects* (2002), and is recently updated in their revised guideline, which states that research designed to obtain knowledge relevant to the health needs of pregnant women must be promoted.^11^ Furthermore, the Second Wave Initiative was launched in 2009; this is a collaborative academic initiative to find ethically and scientifically responsible solutions to increase the knowledge base for the treatment of pregnant women with medical illness.^12^

However, despite all efforts to include pregnant women in research, they are still under‐represented. A 2011 study on all medications approved by the FDA from 1980 to 2010 found that 91% of the medications approved for use by adults did not have sufficient data on safety, efficacy and fetal risk if taken during pregnancy.^13^ At the same time, the number of pregnant women taking medications and the number of medications have both increased. The total percentage of pregnant women who take medications including off‐label medications may currently be as high as 84–99%^14^ and the number of pregnant women taking four or more medications more than tripled over the last three decades, common ones being antibiotics, asthma medications and anti‐nausea medications.^15^ Although these data reinforce the need to study safety and efficacy of drugs in pregnant women specifically, pregnant women generally remain excluded from research. To illustrate, exclusion of pregnant women is common practice in industry‐sponsored phase IV research.^16^ Moreover, a 2014 review demonstrates that between 1960 and 2013, within the pharmacokinetic clinical trials found, less than 1.29% were conducted for pregnant women, and the ones that were undertaken had a strong focus on acute labor and delivery issues.^17^

For a population that is under‐represented in research, it is at least of relevance to understand what ethical boundaries are set by international ethics guidance documents and how and when these documents impede or promote research with pregnant women. Since this *Bioethics Special Issue* focuses on the recently revised CIOMS international ethical guidelines, in this paper we will limit our scope to the CIOMS guidelines.^18^ We will analyse how these guidelines may help to break down the status quo pertaining to the evidence base and hence contribute to fair inclusion of pregnant women in research.

## 2 FAIR INCLUSION AND THE CIOMS GUIDELINES

At least three aspects of the revised CIOMS guidelines may affect fair inclusion of pregnant women in research.

### 2.1 Pregnant women are not vulnerable per se

Unlike some other guidance documents, the CIOMS guidelines never associated pregnant women with vulnerability. Yet, they have shifted their position on inclusion of pregnant women in research from restrictive to permissive. In 1993 the guidelines stated:

As a general rule, pregnant or nursing women should not be subjects of any clinical trials except such trials as are designed to protect or advance the health of pregnant or nursing women or fetuses or nursing infants, and for which women who are not pregnant or nursing would not be suitable subjects.

The 2002 version formed a direct breach with the 1993 version: ‘pregnant women should be presumed to be eligible for participation in biomedical research’. Most likely, this phrase was a direct echo of the claim of the United States’ Office of Research on Women’s Health (ORWH) of the Department of Health and Human Services (DHHS), which stated in 1994 that ‘pregnant women are to be presumed eligible for participation in clinical research’.

This ‘eligibility claim’ of 2002 can be seen as conveying the message of non‐discrimination or equity. However, in the 2016 version, the non‐discrimination underlies Guideline 15 on vulnerable persons and groups. It is no longer present in the guideline on pregnant women as such (Guideline 19). Guideline 15 claims that ‘pregnant women must not be considered vulnerable simply because they are pregnant’. The 2016 version is then the first version to claim that pregnancy itself is not a reason to consider pregnant women as vulnerable and hence not a reason to install special protections. Instead, Guideline 15 claims that special protections may have to be installed depending on the risks to the fetus.

Shifting the underlying rationale of non‐discrimination from the pregnancy guideline to the guideline on vulnerability (Guideline 15) can be explained by the extensive rewrite of the vulnerability guideline itself. The vulnerability guideline no longer classifies entire groups of people as vulnerable, but instead asks researchers and research ethics committees (RECs) to look at characteristics of a person or group that may render the person or group vulnerable and to install special protections tailored to mitigate the risks associated with these characteristics.^19^ Pregnancy per se is no such morally relevant characteristic. Recent literature substantiates the non‐vulnerability statement.^20^ Moreover, even the Common Rule, generally perceived as a more conservative document due to its classification of pregnant women as vulnerable, has changed its position and eliminates the qualification of pregnant women as vulnerable as of July 2018.^21^ In sum, CIOMS’ explicit claim to not regard pregnant women as vulnerable may help to strengthen research into the health needs of pregnant women.

### 2.2 Promote research relevant to the health needs of pregnant women

The CIOMS guidelines now claim that ‘research designed to obtain knowledge relevant to the health needs of the pregnant and breastfeeding woman must be promoted’ (Guideline 19). Analogous to the guidelines on children and adolescents (Guideline 17) and on individuals incapable of giving informed consent (Guideline 16), Guideline 19 also recognizes that pregnant and breastfeeding women have distinctive physiologies and health needs. However, from the idea that distinctive health needs warrant research in this population, it does not follow in Guideline 19 that they must be included in health‐related research unless a good scientific reason justifies their exclusion. In other words, unlike several bioethicists,^22^ the CIOMS guidelines do not go so far that *routine* inclusion of pregnant women in research must be promoted, nor that the default position is to include them unless there is a scientific reason to justify exclusion.

At first sight, the CIOMS guidelines may seem to be inconsistent in Guideline 19, since it does not require justifications of exclusion as written in the guidelines on children and incompetents. At the same time, from a methodological viewpoint, the formulation to promote research may function as a stronger (positive) protection of the health interests of pregnant women and fetuses. Since data on medication use in pregnant women is so scarce, pregnant women will virtually always substantially differ from other populations like children and incompetents. Methodologically, it makes no sense to add a few pregnant women to a population that primarily exists of non‐pregnant women when the factor pregnancy is not taken into account. In those cases, trials should either be conducted separately in pregnant women, or a larger trial should be designed with prespecified subgroup analyses that studies the effects of the intervention in the two groups of women separately.^23^ Moreover, if we know or assume that intervention effects in pregnant women and non‐pregnant women will differ, then an estimated overall effect is not informative since it does not apply to non‐pregnant women or pregnant women, only to a population with a similar distribution of these subgroups.^24^

In other words, ‘promotion of research on pregnant women’ is a stronger protection than having to justify their exclusion. On the one hand, pregnant women are not unnecessarily included in trials that do or cannot take the pregnancy factor into account in the analysis. On the other hand, the health interests of pregnant women are encouraged to be taken into account at a much earlier stage than at the moment of REC review only. The requirement to justify exclusion typically addresses researchers and RECs, respectively with regard to designing studies with pregnant women or reviewing protocols. However, when a researcher applies for funding, ethical (self) assessments are not regularly part of the submission process and researchers may miss that other (sub)groups, such as pregnant women, should have been included in their proposal. Similarly, at the moment of REC review, it is often too late to require researchers to set up separate trials for pregnant women, since researchers have already been funded.

Guideline 19 does not address the typical actors of researcher and REC; instead, the guideline has no specific addressee, unlike most other guidelines in the CIOMS document. The 2016 guideline acts on the level of agenda and priority setting rather than protection by RECs only.

An open issue with regard to the inclusion of pregnant women in research is the timing of inclusion. Elsewhere, we have argued that inclusion of pregnant women in the earliest phases of research can be ethically acceptable, but that preclinical and early clinical data gained in the non‐pregnant population must be available and adequate and that alternative design options must have been considered before pregnant women are included in phase I–II research, such as embedded or adaptive trial designs.^25^ For phase III research, we have argued earlier that these trials should be preceded by phase I–II research and should have been established to a sufficient extent in a non‐pregnant population, before exposing larger numbers of pregnant women, so as to avoid exposure to a potentially non‐effective drug.^26^ Moreover, we have argued that it also matters in phase III that the default position of assuming differences between pregnant and non‐pregnant populations comes down to promoting the set‐up of separate trials or the set‐up of larger trials with prespecified subgroup analyses.^27^ The claim of having to justify exclusion does little to promote these separate trials in pregnant women, since it can only passively review what has been submitted. Instead, the formulation of promoting research relevant to pregnant women’s health needs leaves room for stimulating the set‐up of separate trials, or even the set‐up of registries or more preclinical research that may eventually benefit pregnant women.

In sum, the claim that research relevant to the health needs of pregnant women must be promoted focuses on a broader group of stakeholders to protect the health interests of pregnant women than protection by RECs alone.

### 2.3 Clarified levels of acceptable research risks

In the 2002 version of the CIOMS guidelines the level of acceptable research risks for pregnant women and fetuses remained unclear.^28^ The guideline left it up to pregnant women themselves to decide whether research risks were acceptable: ‘the decision about acceptability of risk to the fetus should be made by the woman as part of the informed consent process’. In 2016, the risk threshold has been clarified and reads as follows:

For research interventions or procedures that have the potential to benefit either pregnant or breastfeeding women or their fetus or infant, risks must be minimized and outweighed by the prospect of potential individual benefit.

For research interventions or procedures that have no potential individual benefits for pregnant and breastfeeding women:

‐ the risks must be minimized and no more than minimal; and

‐ the purpose of the research must be to obtain knowledge relevant to the particular health needs of pregnant or breastfeeding women or their fetuses or infants.

When the social value of the research for pregnant or breastfeeding women or their fetus or infant is compelling, and the research cannot be conducted in non‐pregnant or non‐breastfeeding women, a research ethics committee may permit a minor increase above minimal risk.

The 2016 position on acceptable levels of research risk is similar to what CIOMS deems acceptable for children and persons incapable of giving informed consent. Some will argue that the risk threshold is too weak, since they favor minimal risk thresholds for pregnant women in all forms of research, also in potentially beneficial research.^29^ Others will argue that this standard is too stringent, since pregnant women are capable of giving informed consent and risk thresholds for children and those incapable of giving informed should therefore not directly apply to pregnant women. In addition, advocates of the latter viewpoint may also argue that this risk threshold may withhold pregnant women from participating in a study of high potential benefit to the fetus where the risk to the woman is not minimal, for instance in the case of fetal surgery.

Furthermore, the 2016 CIOMS guidelines do not a priori impede research with high research risks such as fetal surgery. The lines on research that are potentially beneficial (‘For research interventions or procedures that have the potential to benefit either pregnant or breastfeeding women or their fetus or infant, risks must be minimized and outweighed by the prospect of potential individual benefit’) are broad enough to justify fetal surgery that is potentially beneficial. The guidelines do not distinguish between the maternal–fetal entity (which is in line with Blehar and colleagues who argue that pregnant women are a ‘scientifically complex’ group and that the distinction between the woman and the fetus is a ‘false dichotomy’).^30^ In other words, what is a net potential individual benefit for both the woman and the fetus is open for discussion in an REC. Furthermore, consistent with Guideline 4 on research risks and potential benefits, we argue that the surgery should be able to go forward if:

1. The risks to the fetus are outweighed by the potential benefits to the fetus and the woman. Even if the risks to the woman are high, the net risks for both the woman and the fetus may be regarded as acceptable in light of the direct physical benefits to be expected for both the woman and the fetus.
2. The risks to the woman are reasonable in relation to the potential benefits for the fetus and the social value of the research.
3. The risks to the woman respect some upper risk limits that researchers and RECs should further specify in concrete cases. Analogies that RECs may take into account are comparable upper risk thresholds in the case of altruistic organ donation and research with competent consenting participants.

In sum, the now clarified threshold for acceptable research risks may help to prevent pregnant women being unnecessarily excluded from research.

## 3 DISCUSSION

The 2016 CIOMS guidelines contribute to fair inclusion of pregnant women in research to the extent that pregnant women are explicitly not seen as vulnerable, research relevant to their health needs is promoted, and acceptable levels of risk have been clarified. Moreover, since pregnant women are still under‐represented in research and the status quo in knowledge generation for medications and treatments of pregnant women is substantial, it seems justified to maintain a separate guideline on the interests of pregnant women as research participants.

Evidently, fair inclusion of pregnant women will not be achieved by clarification of guidance documents alone. For instance, while guidelines such as CIOMS have widened the possibility for research with pregnant women, interpretation and perception of such documents is something different. Mechanisms of over‐ and underprotection play a role. An example of overprotection is that healthcare professionals may be reluctant to include pregnant women because they perceive a study to be high risk, even though an REC classifies the study as low risk.^31^ Underprotection also applies in the sense that research and care have become totally distinct worlds in the field of drug use in pregnancy. Paradoxically, pregnant women are excluded from research for protection, while they run high risks in routine practice, yet without learning from this exposure to protect future medication users. A potential solution for both over‐ and underprotection of pregnant women taking medications during pregnancy is to transform the field into a so‐called learning health system (LHS). As we argued above, since CIOMS Guideline 19 is structured as a guideline that is primarily focused on priority and agenda setting, it allows for paradigm changes in knowledge generation, such as transformation to an LHS.

In 2006, the U.S. Institute of Medicine called on healthcare leaders to transform their health systems into ‘learning health systems’, meaning a system that is designed to generate and apply the best evidence for the collaborative healthcare choices of each patient and provider; to drive the process of discovery as a natural outgrowth of patient care; and to ensure innovation, quality, safety, and value in healthcare.^32^ The idea of transforming health practices to LHSs increasingly attracts attention, but remains a rather theoretical exercise. It has primarily been discussed in the context of quality improvement and comparative effectiveness research,^33^ but there are still many open issues in LHS theory, including ethics and methods.^34^ Moreover, it has thus far not been proposed to solve the status quo in knowledge generation for medication use in pregnancy.

An LHS for knowledge generation in the field of medication use of pregnant women is a promising way forward since it no longer sharply distinguishes between research and care. Instead, it acknowledges that the care context should also be used to learn and should be governed by ethical oversight at the same time, since pregnant women currently de facto participate in n‐of‐1 trials without protection and learning for future generations. Moreover, it acknowledges that the traditional way of knowledge generation is not ideally suited to maximize the desired output. Formal randomized controlled trials (RCTs) with pregnant women who use drugs, in particular for non‐pregnancy‐related indications, are difficult to perform. Many argue that these trials can only start after a trial in the non‐pregnant population has been performed. Often, this approach results in a situation that no such trial in pregnant women is performed.^35^ Furthermore, long‐term follow‐up of neonates is usually essential to reveal drug‐related risks. Also, RCTs are often monodisciplinary, where research outcomes and risks for the mother and future child should be considered multidisciplinary (obstetricians, pediatricians and others, such as rheumatologists and psychiatrists) to optimally determine safety and efficacy of these drugs. Therefore, methods other than RCTs are of utmost importance to speed up the knowledge generation in this field. Despite the availability of promising alternatives, such as registry‐based studies, the current research infrastructure does not allow for these alternatives, since solid and comprehensive registries combined with pregnancy‐tailored trial methodology are lacking. Moreover, there is limited insight into the advantages and disadvantages of alternative designs in the field of medication use in pregnancy.^36^

Another crucial factor to realize transformation to an LHS is multi‐stakeholder involvement. Pharma, physicians, patients, funders, regulators and others should all support the idea that we have to leave the current paradigm of knowledge generation in the field of pregnancy. Since an LHS drives on co‐creation, meaning that scientists, clinicians, patients, and others collectively generate new knowledge in a dynamic interaction,^37^ the LHS seems a promising way forward to realize the transformation. Co‐creation implies that stakeholder involvement is essential to further health knowledge that leads to meaningful outcomes, for instance by co‐deciding on research questions, methods, endpoints and dissemination of results.

In order to transform the field of pregnancy into an LHS, we think that the following features are essential. First, we have to create an, ideally global, meta‐registry. Currently, several databases, cohorts and registries exist with unique data regarding pregnancy, adverse drug reactions and the like, such as EUROmediCAT^38^ and ENTIS.^39^ However, these data sources are not designed to combine data on the drug used, endpoints to determine efficacy and safety of the drug used to treat, birth defects and potential long‐term consequences of use of the specific medicines during pregnancy. This meta‐registry is essential for clinical practice, since physicians and women are not only interested in teratogenicity, but also in adverse maternal and fetal events, related to either using or not using a drug. For instance, the risk on teratogenicity should be weighed against risks of serious infections for the neonate when medication is discontinued. Evidently, there is a myriad of challenges regarding the development and implementation of a global meta‐registry, such as the linking of data of pregnant women to those of the fetus and child and differences across registries in terms of terminology, formatting, and labelling. For managerial reasons it is therefore reasonable to start small and accordingly publish the results and share them more broadly.

Second, we have to develop appropriate methodology to gather knowledge in the field of pregnancy. Conventional RCTs are typically difficult to perform in pregnant women. Because of additional fetal risks associated with including pregnant women in clinical research and the altered ways in which drugs are processed by the pregnant body, pregnant women cannot be treated as an ordinary subgroup in the various phases of traditional drug development. A meta‐registry creates an opportunity to allow for alternative research designs that are able to capture long‐term follow‐up data, for instance registry‐based RCTs and Bayesian designs. However, literature on appropriate methodology for research in pregnant women is scarce and potential research designs profoundly need further study and evaluation.^40^

Third, we have to take a co‐creationistic approach. One explanation for the continuing status quo is the lack of ownership of the problem.^41^ There is a variety of stakeholders related to research with pregnant women, such as fertile and pregnant women themselves, investigators, Pharma, healthcare professionals, patients, regulators, journal editors, pharmacovigilance centers and others. At the moment, all stakeholders bear partial responsibility, but none of these stakeholders is *ultimately* responsible for improving the evidence base for medication use in pregnant women.

It has been argued that fair inclusion at least requires a concerted effort to change the status quo.^42^ Eventually, acceptability of the shared ownership problem of health inequity of pregnant women is essential to enforce a breakthrough in knowledge generation of medication use for (chronically) ill pregnant women. We cannot and should not wait for pregnant women themselves to seize this opportunity, as the temporary nature of pregnancy renders this situation highly unlikely.

In sum, transformation to an LHS should be explored to learn from routinely collected data instead of unilateral insistence on more RCTs in pregnant women to break down the status quo in knowledge generation in this field. CIOMS Guideline 19 allows for this transformation process.

## 4 CONCLUSION

The CIOMS guidelines of 2016 have been revised in ways that may help to break down the status quo in knowledge generation on drugs and treatments for pregnant women and hence promote the fair inclusion of pregnant women in research. Moreover, the structure of Guideline 19 on pregnant women provides a unique opportunity to realize a transformation of the field of medication use in pregnancy to an LHS, which is essential to advance this field.

## CONFLICT OF INTEREST

The authors declare no conflict of interest.

## Notes

1 Allesee, L., & Gallagher, C. M. (2011). Pregnancy and protection: The ethics of limiting a pregnant woman’s participation in clinical trials. *Journal of Clinical Research & Bioethics*,* 2*(108), pii: 1000108; Swan, S. H. (2000). Intrauterine exposure to diethylstilbestrol: Long‐term effects in humans. *APMIS*,* 108*, 793–804.

2 Macklin, R. (2010). Enrolling pregnant women in biomedical research. *Lancet*,* 375*, 632–633.

3 Langston, L. (2016). Better safe than sorry: Risk, stigma and research during pregnancy. In F. Baylis & A. Ballantyne (Eds.), *Clinical research involving pregnant women* (pp. 1–8). Cham, Switzerland: Springer International Publishing.

4 Baylis, F. (2010). Pregnant women deserve better. *Nature, 465*, 689–690.

5 Baylis, F., & MacQuarrie, R. (2016). Why physicians and women should want pregnant women included in clinical trials. In F. Baylis & A. Ballantyne A. (Eds.), *Clinical research involving pregnant women* (pp. 17–32). Cham, Switzerland: Springer International Publishing.

6 Molenaar, N. M., Brouwer, M. E., Duvekot, J. J., Burger, H., Knijff, E. M., Hoogendijk, W. J. … Lambregtse‐van den Berg, M. P. (2018). Antidepressants during pregnancy: Guideline adherence and current practice amongst Dutch gynaecologists and midwives. *Midwifery*,* 61*, 29–35.

7 Little, M., Lyerly, A., & Faden, R. (2009). Pregnant women and medical research: A moral imperative. *Bioethica Forum*,* 2*, 60–65; Buhimschi, C. S., & Weiner, C. P. (2009). Medications in pregnancy and lactation: Part 1. Teratology. *Obstetrics & Gynecology*,* 113*, 166–188; McCormack, S., & Best, B. M. (2014). Obstetric pharmacokinetic dosing studies are urgently needed. *Frontiers in Pediatrics*,* 2*, 9.

8 Little et al., *op. cit*. note 7; Lyerly, A. D., Little, M. O., & Faden, R. (2008). The second wave: Toward responsible inclusion of pregnant women in research. *International Journal of Feminist Approaches to Bioethics*,* 1*, 5–22.

9 Mazer‐Amirshahi, M., Samiee‐Zafarghandy, S., Gray, G., & van den Anker, J. N. (2014). Trends in pregnancy labeling and data quality for US‐approved pharmaceuticals. *American Journal of Obstetrics & Gynecology*,* 211*, 690.e1–11; United States Department of Health and Human Services. (2005). *Reviewer guidance: Evaluating the risks of drug exposure in human pregnancies*. Rockville, MD: Office of Training and Communications.

10 Little, B. B. (1999). Pharmacokinetics during pregnancy: Evidence‐based maternal dose formulation. *Obstetrics & Gynecology*,* 93*, 858–868; Blehar, M. C., Spong, C., Grady, C., Goldkind, S. F., Sahin, L., & Clayton, J. A. (2013). Enrolling pregnant women: Issues in clinical research. *Women’s Health Issues*,* 23*, e39–e45; Chambers, C. D., Polifka, J. E., & Friedman, J. M. (2008). Drug safety in pregnant women and their babies: Ignorance not bliss. *Clinical Pharmacology & Therapeutics*,* 83*, 181–183; Sheffield, J. S., Siegel, D., Mirochnick, M., Heine, R. P., Nguyen, C., Bergman, K. L., … Nesin, M. (2014). Designing drug trials: Considerations for pregnant women. *Clinical Infectious Diseases*,* 59*, S437–444; Haas, D. M., Gallauresi, B., Shields, K., Zeitlin, D., Clark, S. M., Hebert, M. F., … Renbarger J. L. (2011). Pharmacotherapy and pregnancy: Highlights from the third international conference for individualized pharmacotherapy in pregnancy. *Clinical & Translational Science*,* 4*, 204–209.

11 Council for International Organizations of Medical Sciences (CIOMS). (2016)*. International ethical guidelines for health‐related research involving humans*. Geneva, Switzerland: CIOMS.

12 Little, Lyerly & Faden, *op. cit*. note 8; The Second Wave Initiative. Retrieved from https://secondwaveinitiative.org/

13 Adam, M. P., Polifka, J. E., & Friedman, J. M. (2011). Evolving knowledge of the teratogenicity of medications in human pregnancy. *American Journal of Medical Genetics Part C: Seminars in Medical Genetics*,* 157C*, 175–182.

14 Second Wave Initiative, *op. cit*. note 12; European Medicines Agency (EMA). (2007). *Reflection paper on methodological issues in confirmatory clinical trials planned with an adaptive design. Reproduction*. London, UK: EMA; Noah, B. A. (2014). The inclusion of pregnant women in clinical research. *Faculty Publication Western New England University School of Law*, 353–389; Lupattelli, A., Spigset, O., Twigg, M. J., Zagorodnikova, K., Mårdby, A. C., Moretti, M. E., … Nordengl, H. (2014). Medication use in pregnancy: A cross‐sectional, multinational web‐based study. *BMJ Open*,* 4*, e004365; Mitchell, A. A., Gilboa, S. M., Werler, M. M., Kelley, K. E., Louik, C., & Hernández‐Díaz, S. (2011). Medication use during pregnancy, with particular focus on prescription drugs: 1976–2008. *American Journal of Obstetrics & Gynecology*,* 205*(51), e1–e8.

15 McCormack & Best, *op. cit*. note 7; Parisi, M. A., Spong, C. Y., Zajicek, A., & Guttmacher, A. E. (2011). We don’t know what we don’t study: The case for research on medication effects in pregnancy. *American Journal of Medical Genetics Part C: Seminars in Medical Genetics*,* 157*, 247–250.

16 Shields, K. E., & Lyerly, A. D. (2013). Exclusion of pregnant women from industry‐sponsored clinical trials. *Obstetrics & Gynecology*,* 122*, 1077–1081; Scaffidi, J., Mol, B., & Keelan, J. (2016). The pregnant women as a drug orphan: A global survey of registered clinical trials of pharmacological interventions in pregnancy. *BJOG*,* 124*, 132–140.

17 McCormack & Best, *op. cit*. note 7.

18 CIOMS, *op. cit*. note 11.

19 Luna, F., & Vanderpoel, S. (2013). Not the usual suspects: Addressing layers of vulnerability. *Bioethics*,* 27*, 325–332.

20 van der Zande, I. S. E., van der Graaf, R., Oudijk, M. A., & van Delden, J. J. M. (2017). Vulnerability of pregnant women in research. *Journal of Medical Ethics*,* 43*(10), 657–663; Krubiner, C. B., & Faden, R. R. (2017). Pregnant women should not be categorised as a 'vulnerable population' in biomedical research studies: Ending a vicious cycle of 'vulnerability'. *Journal of Medical Ethics*,* 43*(10), 664–665.

21 Office for Human Research Protections. (2018). *Revised Common Rule*. Rockville, MD: Office for Human Research Protections.

22 Kaposy, C. (2016). Presumptive inclusion and legitimate exclusion criteria. In F. Baylis & A. Ballantyne (Eds.), *Clinical research involving pregnant women* (pp. 51–62). Cham, Switzerland: Springer International Publishing; Baylis, F., & Halperin, S. A. (2012). Research involving pregnant women: Trials and tribulations. *Clinical Investigation*,* 2*(2), 139–146.

23 van der Graaf, R., van der Zande, I. S. E., den Ruijter, H. M., Oudijk, M. A., van Delden, J. J. M., Oude Rengerink, K., & Groenwold, R. H. H. (2018). Fair inclusion of pregnant women in clinical trials: An integrated scientific and ethical approach. *Trials*,* 19*(1), 78.

24 Ibid.

25 Baylis & Halperin *op. cit*. note 22; Roes, K. C. B., van der Zande, I. S. E., van Smeden, M., & van der Graaf, R. (2018). Towards an appropriate framework to facilitate responsible inclusion of pregnant women in drug development programs. *Trials*,* 19*, 123.

26 Ibid.

27 van der Graaf et al., *op. cit*. note 23.

28 Kaposy, *op. cit*. note 22, p. 56.

29 van der Zande, I. S. E., van der Graaf, R., Oudijk, M. A., & van Delden, J. J. M. (2017). A qualitative study on acceptable levels of risk for pregnant women in clinical research. *BMC Medical Ethics*,* 18*(1), 35.

30 Blehar et al., *op. cit*. note 10.

31 van der Zande et al., *op. cit*. note 29.

32 Institute of Medicine (IOM). (2007). Roundtable on evidence‐based medicine, the learning healthcare system: Workshop summary, L. Olsen, D. Aisner, & J. M. McGinnis (Eds.). Washington, DC: National Academies Press. Retrieved from https://www.nationalacademies.org/hmd/reports/2007/the-learning-healthcare-system-workshop-summary.aspx; Budrionis, A., & Bellika, J. G. (2016). The learning healthcare system: Where are we now? A systematic review*. Journal of Biomedical Informatics*,* 64*, 87–92.

33 Faden, R. R., Kass, N. E., Goodman, S. N., Pronovost, P., Tunis, S., & Beauchamp, T. L. (2013). An ethics framework for a learning health care system: A departure from traditional research ethics and clinical ethics. *Hastings Center Report*,* 43*, S16–S27.

34 Budrionis & Bellika, *op. cit*. note 32; Faden et al., *op. cit*. note 33.

35 Baylis, *op. cit*. note 4; Baylis & MacQuarrie, *op. cit*. note 5; Little et al., *op. cit*. note 7.

36 Baylis & Halperin, *op. cit*. note 22.

37 Jasanoff, S. (2016). *States of knowledge: The co‐production of science and the social order*. London, UK: Routledge.

38 EUROmediCat. Retrieved from https://www.euromedicat.eu/whatiseuromedicat

39 European Network of Teratology Information Services (ENTIS). Retrieved from https://www.entis-org.eu/

40 Roes et al., *op. cit*. note 25.

41 Van der Graaf et al., *op. cit*. note 23.

42 Ibid.

